# Immortalized Canine Adipose-Derived Mesenchymal Stem Cells Maintain the Immunomodulatory Capacity of the Original Primary Cells

**DOI:** 10.3390/ijms242417484

**Published:** 2023-12-14

**Authors:** Yuyo Yasumura, Takahiro Teshima, Tomokazu Nagashima, Masaki Michishita, Takashi Takano, Yoshiaki Taira, Ryohei Suzuki, Hirotaka Matsumoto

**Affiliations:** 1 Laboratory of Veterinary Internal Medicine, Department of Veterinary Clinical Medicine, School of Veterinary Medicine, Faculty of Veterinary Science, Nippon Veterinary and Life Science University, 1-7-1 Kyonan-cho, Musashino, Tokyo 180-8602, Japan; d2203@nvlu.ac.jp (Y.Y.); v18039@nvlu.ac.jp (Y.T.); ryoheisuzuki@nvlu.ac.jp (R.S.); matsumoto@nvlu.ac.jp (H.M.); 2 Research Center for Animal Life Science, Nippon Veterinary and Life Science University, 1-7-1 Kyonan-cho, Musashino, Tokyo 180-8602, Japan; 3 Laboratory of Veterinary Pathology, Department of Veterinary Clinical Medicine, School of Veterinary Medicine, Faculty of Veterinary Science, Nippon Veterinary and Life Science University, 1-7-1 Kyonan-cho, Musashino, Tokyo 180-8602, Japan; d2202@nvlu.ac.jp (T.N.); michishita@nvlu.ac.jp (M.M.); 4 Laboratory of Veterinary Public Health, Department of Veterinary Clinical Medicine, School of Veterinary Medicine, Faculty of Veterinary Science, Nippon Veterinary and Life Science University, 1-7-1 Kyonan-cho, Musashino, Tokyo 180-8602, Japan; ttakano@nvlu.ac.jp

**Keywords:** immunomodulation, dog, mesenchymal stem cell, immortalization, DSS-induced colitis, macrophage, lymphocyte

## Abstract

Mesenchymal stem cells (MSCs) are a promising cell source for stem cell therapy of intractable diseases in veterinary medicine, but donor-dependent cellular heterogeneity is an issue that influences therapeutic efficacy. Thus, we previously established immortalized cells that maintain the fundamental properties of primary cells, but functional evaluation had not been performed. Therefore, we evaluated the immunomodulatory capacity of the immortalized canine adipose-derived MSCs (cADSCs) in vitro and in vivo to investigate whether they maintain primary cell functions. C57BL/6J mice were treated with dextran sulfate sodium (DSS) to induce colitis, injected intraperitoneally with immortalized or primary cADSCs on day 2 of DSS treatment, and observed for 10 days. Administration of immortalized cADSCs improved body weight loss and the disease activity index (DAI) in DSS-induced colitic mice by shifting peritoneal macrophage polarity from the M1 to M2 phenotype, suppressing T helper (Th) 1/Th17 cell responses and inducing regulatory T (Treg) cells. They also inhibited the proliferation of mouse and canine T cells in vitro. These immunomodulatory effects were comparable with primary cells. These results highlight the feasibility of our immortalized cADSCs as a cell source for stem cell therapy with stable therapeutic efficacy because they maintain the immunomodulatory capacity of primary cells.

## 1. Introduction

Mesenchymal stem cells (MSCs) are a cell population that exist as perivascular cells in most tissues and act to maintain tissue homeostasis through stromal remodeling and immunomodulatory actions [1,2]. Because of the multimodal immunomodulatory and anti-inflammatory effects of MSCs mediated by secreted factors, such as cytokines, chemokines, growth factors, exosomes, and microRNAs, MSC-based stem cell therapy has been proposed to be a new treatment option for immune-mediated and inflammatory diseases that do not respond to existing standard treatments, such as immunosuppressive drugs [3,4,5]. In fact, many clinical studies have been conducted in animals and humans, and some have reported promising results [6,7]. However, some results from multiple clinical studies of MSCs contradict the results of in vitro and in vivo preclinical studies [8,9]. MSCs are a heterogeneous cell population with a mixture of cells that exhibit physiological functions expected from the perspective of disease treatment as well as cells that do not exhibit such physiological functions [10,11]. MSC properties vary depending on the donor, tissue source, and manufacturing methods such as isolation, expansion, and freezing [12,13]. It is possible that this heterogeneity of MSCs, which creates instability in multiple secreted factors, is a major cause of variability in the results of clinical trials [9,10]. Although tissue sources and manufacturing methods can be limited to reduce MSC variability and stabilize therapeutic effects, it is impossible to limit donors because primary MSCs have finite proliferation and will cease to proliferate with continued passaging because of the cell lifespan. Therefore, we previously established immortalized canine adipose-derived MSCs (cADSCs) by transfection of primary cADSCs with a combination of human R24C mutant cyclin-dependent kinase (*CDK4R24C*), canine cyclin D1 (*CCND1*), and/or canine telomerase reverse transcriptase (*TERT*) [14]. This method has been reported to be able to immortalize primary cells in many species while preserving their properties [15,16]. Our immortalized cADSCs maintained the fundamental characteristics of primary cADSCs in terms of cell surface marker expression patterns and trilineage differentiation potential, had no chromosomal abnormalities or tumorigenic potential, and continued to proliferate without cell senescence [14]. Thus, immortalized cADSCs are a promising cell source for research with high reproducibility and stem cell therapy with stable efficacy, but further investigation is needed to determine whether they maintain the functional characteristics of primary cADSCs.

MSCs were initially studied mainly because of their potential to repair and regenerate tissue [17]. However, MSCs are now being actively investigated for applications in therapies against immune-mediated diseases, such as acute graft-versus-host disease (GVHD), because of their anti-inflammatory effects mediated by suppressing excessive immune responses and low risk of causing further immune responses upon administration owing to their low immunogenicity [3,18,19]. MSCs modulate both innate and adaptive immune responses by interacting with various immune cell types, including T cells [20,21], B cells [22], natural killer cells [23], dendritic cells [24], macrophages/monocytes [25,26], and neutrophils [27]. Among them, the interactions with T cells and macrophages play a particularly important role in the immunomodulatory actions of MSCs, and numerous studies have reported that MSCs exert their therapeutic effects by altering the phenotype and function of these cells [18,28]. It has also been suggested that MSCs alter their immunomodulatory effects in response to the disease status and local microenvironment [29,30]. Indeed, when stimulated with inflammatory cytokines or co-cultured with immune cells, the profile of secreted factors from MSCs shows marked changes [31]. However, these settings do not accurately mimic the inflammatory environment in vivo. Thus, the immunomodulatory potential of MSCs must be evaluated both in vitro and in vivo [32,33]. Therefore, in this study, we evaluated whether immortalized cADSCs maintain the functional properties of primary cADSCs by analyzing the immunomodulatory capacity of immortalized cADSCs in vitro and in vivo using a mouse model of dextran sulfate sodium (DSS)-induced colitis, which is commonly used in MSC research.

## 2. Results

### 2.1. Therapeutic Effect of Immortalized cADSCs Comparable with Primary cADSCs on DSS-induced Colitis

**Figure 1:**
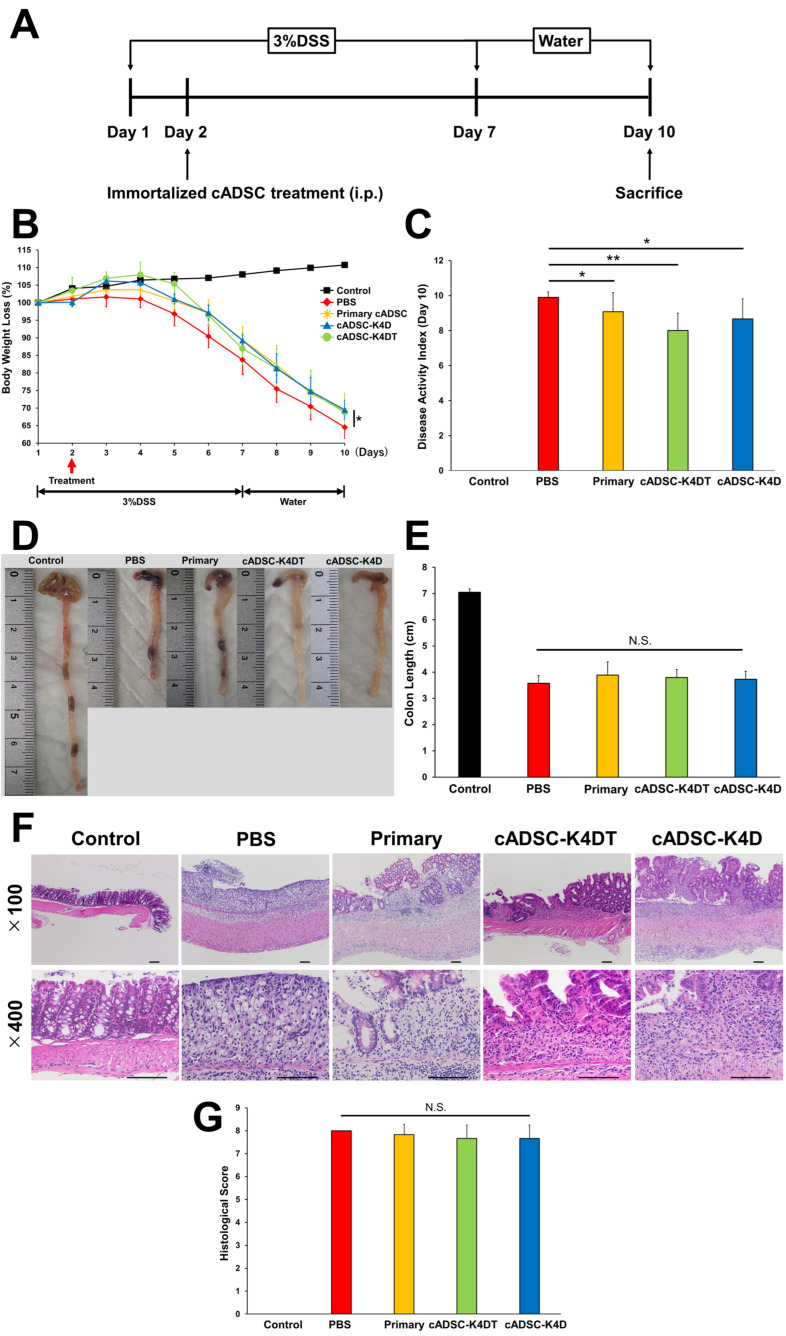
Therapeutic efficacy of immortalized canine adipose-derived mesenchymal stem cells (cADSCs) in dextran surfate sodium (DSS)-induced colitis. (**A**) Scheme for colitis induction by DSS and administration of immortalized cADSCs; (**B**) changes in body weight throughout the experimental period. Body weight loss at day 10 was significantly reduced in cADSC-treated groups compared with the PBS group; (**C**) disease activity index (DAI) at day 10. cADSC-treated groups scored significantly lower than the phosphate buffer saline (PBS)-treated group; (**D**) Pphotographs of the colon of the mice in each group; (**E**) colon shortening was not inhibited by administration of cADSCs; (**F**) histological images of the colon of each group (upper: ×100, lower: ×400, bar = 100 μm); (**G**) histological score of the colon in each group. Data are expressed as the mean ± standard deviation; *n* = 5; * *p* < 0.05, ** *p* < 0.01, N.S. = not statistically significant.

To compare the therapeutic effect of immortalized cADSCs with primary cADSCs on DSS-induced colitis, primary and immortalized cADSCs were injected intraperitoneally into mice at 24 h after colitis induction (Figure 1A). Body weight loss at day 10, the end of the experiment, was slightly but significantly milder in the primary cADSC-treated group (69.3% ± 5.0%) and immortalized cADSC-treated groups (cADSC-K4DT: 68.8% ± 4.8%; cADSC-K4D: 69.5% ± 2.7%) than in the phosphate-buffered saline (PBS)-treated group (64.5% ± 3.2%; Figure 1B). The disease activity index (DAI) was predominantly elevated in DSS-treated mice compared with healthy control mice, whereas mild but significant suppression of the elevation was observed in the primary cADSC-treated group (9.1 ± 1.1) and immortalized cADSC-treated groups (cADSC-K4DT: 8.0 ± 1.0; cADSC-K4D: 8.7 ± 1.2) compared with the PBS-treated group (9.9 ± 0.3; Figure 1B). However, colon length shortening (PBS: 3.6 cm ± 0.3 cm; primary: 3.9 cm ± 0.5 cm; cADSC-K4DT: 3.8 cm ± 0.3 cm; cADSC-K4D: 3.7 cm ± 0.3 cm) and histological scores (PBS: 8.0 ± 0.0; primary: 7.8 ± 0.5; cADSC-K4DT: 7.7 ± 0.6; cADSC-K4D: 7.7 ± 0.6) were not significantly different between the PBS and cADSC groups (Figure 1D–G). As shown in Figure 1F, all DSS-treated mice showed histologically severe inflammatory cell infiltration in the colon, but a relatively mild degree of mucosal epithelial injury was observed in the groups treated with primary or immortalized cADSCs.

### 2.2. Shift in Macrophage Polarity from the M1 to M2 Phenotype In Vivo by Immortalized cADSCs

**Figure 2:**
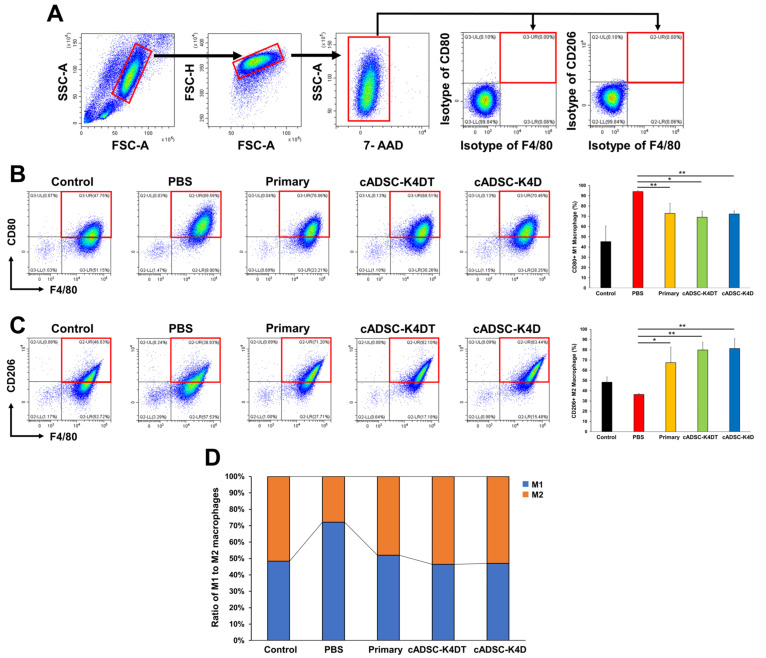
Effect of primary and immortalized cADSCs on the phenotype of peritoneal macrophages in mice with DSS-induced colitis. (**A**) Gating strategy for phenotypic analysis of peritoneal macrophages by flow cytometry. Red boxes indicate gating regions. From the left panel: gating of macrophage fractions, removal of doublets and dead cells, gating of F4/80 and CD80 or CD206 co-positive regions. At least twenty thousand F4/80-positive cells were measured at each experiment; (**B**) F4/80^+^CD80^+^ M1 macrophage frequency determined by flow cytometry. Primary and immortalized cADSCs suppressed the increase in M1 macrophages induced by DSS administration; (**C**) F4/80^+^CD206^+^ M2 macrophage frequency. Primary and immortalized cADSCs increased M2 macrophages in mice with colitis; (**D**) percentages of M1 and M2 phenotypes among total macrophages. Primary and immortalized cADSCs balanced the polarity of macrophages polarized toward M1 dominance. Data are expressed as the mean ± standard deviation; *n* = 5; * *p* < 0.05, ** *p* < 0.01.

To evaluate the immunomodulatory effects of immortalized cADSCs on macrophages, we investigated the phenotype of peritoneal macrophages from mice with DSS-induced colitis treated with primary or immortalized cADSCs. To this end, we selected F4/80 as a cell surface marker for pan-macrophages, CD80 for the proinflammatory phenotype (M1), and CD206 for the anti-inflammatory phenotype (M2), and analyzed the frequency of macrophages with each phenotype. F4/80^+^CD80^+^ M1 macrophages significantly increased in colitic mice without cADSC treatment (94.0% ± 1.0%) compared with healthy control mice (45.3% ± 15.3%), but the increase was significantly suppressed in mice treated with primary cADSCs (72.9% ± 9.6%) or immortalized cADSCs (cADSC-K4DT: 69.2% ± 5.8%; cADSC-K4D: 72.2% ± 3.0%; Figure 2A). Conversely, F4/80^+^CD206^+^ M2 macrophages decreased in colitic mice without cADSC treatment (36.4% ± 0.9%) compared with healthy mice (48.3% ± 5.1%), whereas administration of primary cADSCs (67.4% ± 15.2%) or immortalized cADSCs (cADSC-K4DT: 79.7% ± 7.7%, cADSC-K4D: 81.2% ± 9.7%) significantly increased M2 macrophages in colitic mice (Figure 2B). Among total macrophages, the percentage of M1 or M2 phenotypes was predominantly M1 (approximately 70%) in colitic mice without cADSC treatment and approximately 50% in colitic mice treated with primary cADSCs, cADSC-K4DT, or cADSC-K4D, which was comparable with healthy mice (Figure 2C).

### 2.3. Inhibition of T helper (Th) 1/Th17 Cell Responses and Induction of Treg Cells by Immortalized cADSCs In Vivo

**Figure 3:**
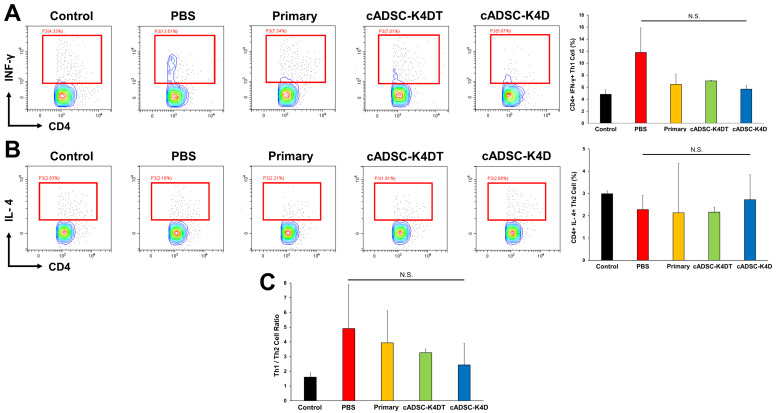
Effect of immortalized cADSCs on T helper (Th)1/Th2 cells. (**A**) CD4^+^IFN-γ^+^ Th1 cell frequency determined by flow cytometry. Immortalized cADSCs suppressed Th1 cell responses activated by DSS treatment as much as primary cADSCs; (**B**) Th2 cell responses were unaffected by administration of primary or immortalized cADSCs; (**C**) ratio of Th1 and Th2 cell frequencies as a measure of the Th1/Th2 cell balance. Primary and immortalized cADSCs modulated the Th1/Th2 cell balance by suppressing Th1 cell responses. Data are expressed as the mean ± standard deviation; *n* = 5; N.S. = not statistically significant.

To assess whether immortalized cADSCs modulate the Th cell paradigm and balance polarized Th cells, we investigated the phenotype of CD4^+^ Th cells derived from the spleen of mice with DSS-induced colitis. To determine the effect of immortalized cADSCs on the balance of Th1/Th2 cells, the classic Th cell type, we stained CD4^+^ Th cells for interferon (IFN)-γ as a Th1 cell marker and IL-4 as a Th2 cell marker and measured the frequency of each positive cell by flow cytometry. CD4^+^IFN-γ^+^ Th1 cells greatly increased in the spleens of DSS-induced colitic mice (11.8% ± 8.7%) compared with healthy control mice (4.8% ± 1.1%), although the difference was not statistically significant, whereas administration of primary cADSCs (6.5% ± 6.2%) and immortalized cADSCs (cADSC-K4DT: 7.0% ± 0.2%; cADSC-K4D: 5.7% ± 1.2%; Figure 3A) suppressed the Th1 cell increase to approximately baseline levels. The frequency of CD4^+^IL-4^+^ Th2 cells was mildly reduced after induction of colitis by DSS, which did not change with or without cADSC administration (control: 3.0% ± 0.1%; PBS: 2.3% ± 0.6%; primary: 2.1% ± 2.2%; cADSC-K4DT: 2.2% ± 0.2%; cADSC-K4D: 2.7% ± 1.1%; Figure 3B). The ratio of Th1 and Th2 cell frequencies as a measure of the Th1/Th2 balance tended to increase in DSS-treated mice, but decreased toward baseline levels in mice that received cADSCs, although the difference was not statistically significant (control: 1.6 ± 0.3; PBS: 4.9 ± 3.0; primary: 3.9 ± 2.2; cADSC-K4DT: 3.3 ± 0.3; cADSC-K4D: 2.4 ± 1.5; Figure 3C).

**Figure 4:**
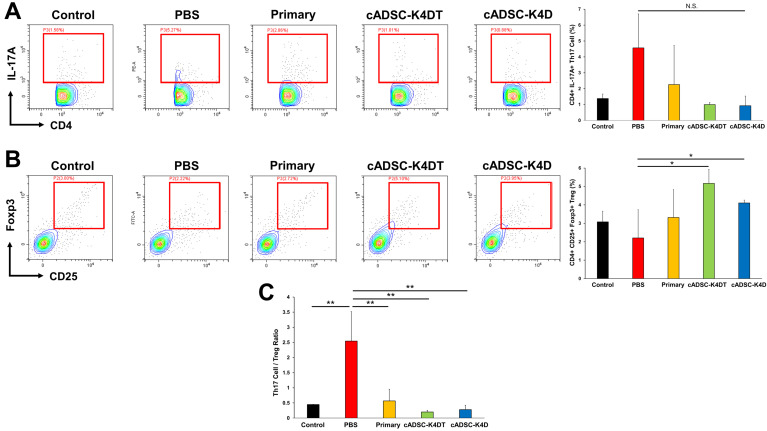
Effect of immortalized cADSCs on Th17/regulatory T (Treg) cells. (**A**) CD4^+^IL-17A^+^ Th17 cell frequency determined by flow cytometry. Immortalized cADSCs suppressed Th17 cell responses activated by DSS treatment more than primary cADSCs; (**B**) immortalized cADSCs increased Treg cells more than primary cADSCs; (**C**) ratio of Th17 and Treg cell frequencies as a measure of the Th17/Treg cell balance. Primary and immortalized cADSCs regulated the Th17/Treg cell balance by suppressing Th17 cell responses and inducing Treg cells. Data are expressed as the mean ± standard deviation; *n* = 5; * *p* < 0.05, ** *p* < 0.01, N.S. = not statistically significant.

Next, we examined the effects of immortalized cADSCs on Th17/regulatory T (Treg) cell-mediated immunity, a major player in the pathogenesis of inflammatory bowel disease (IBD) in humans [34] and mice [35] and an important target for the immunomodulatory actions of MSCs [36]. For this purpose, we examined the frequency of Th17 cells, which are positive for CD4 and express cytokine IL-17A, and Treg cells, which are co-positive for CD4 and CD25 and express nuclear Foxp3, in DSS-induced colitic mice treated with immortalized cADSCs and compared them with mice treated with primary cADSCs or PBS. As a result, Th17 cells clearly increased in DSS-induced colitic mice (4.6% ± 2.1%) compared with healthy control mice (1.4% ± 0.3%), although not statistically significant, and administration of primary cADSCs (2.3% ± 2.5%) and immortalized cADSCs (cADSC-K4DT: 1.0% ± 0.1%; cADSC-K4D: 0.9% ± 0.6%; Figure 4A) suppressed the increase in Th17 cells to near the baseline. Conversely, Treg cells mildly decreased by treatment with DSS (2.2% ± 1.5%) compared with healthy control mice (3.1% ± 0.6%) but recovered to approximately baseline levels after administration of primary cADSCs (3.3% ± 1.5%) and, surprisingly, increased significantly above the baseline after administration of immortalized cADSCs (cADSC-K4DT: 5.2% ± 0.8%; cADSC-K4D: 4.1% ± 0.2%; Figure 4B). Moreover, to assess the ability of immortalized cADSCs to regulate the Th17/Treg cell balance, the frequency ratio of Th17 and Treg cells was calculated and compared in each group. As a result, DSS administration (2.5 ± 1.0) significantly increased the Th17/Treg cell ratio compared with healthy control mice (0.4 ± 0.0), whereas primary cADSCs (0.6 ± 0.4) and immortalized cADSCs (cADSC-K4DT: 0.2 ± 0.1; cADSC-K4D: 0.3 ± 0.1; Figure 4C) significantly decreased the Th17/Treg cell ratio, thereby modulating the Th17/Treg cell balance.

### 2.4. Immortalized cADSCs Inhibit the Proliferation of CD4^+^ Th Cells in Mice with Colitis

**Figure 5:**
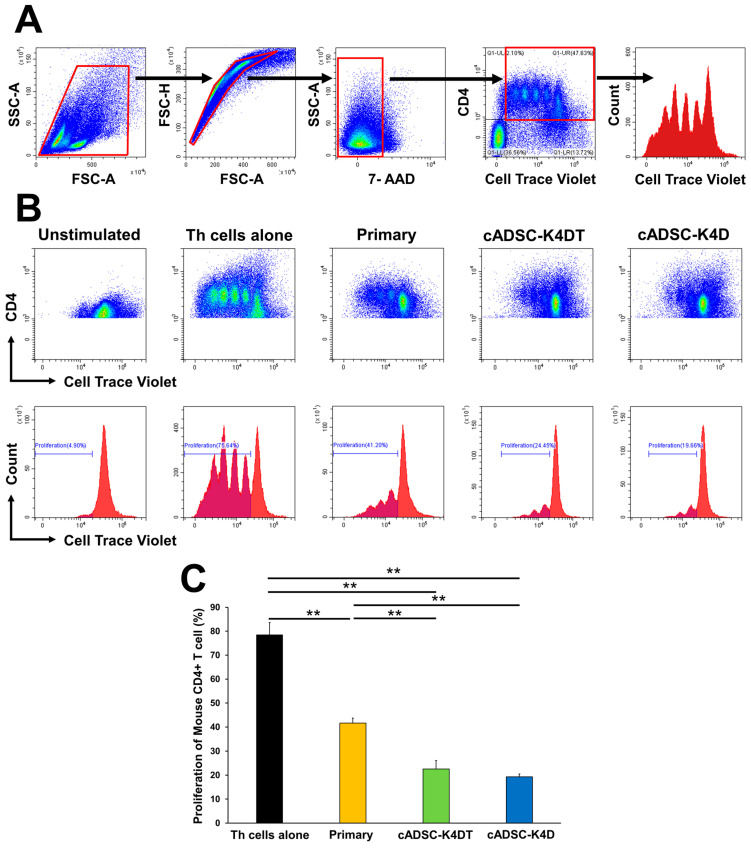
Proliferation inhibitory effect of immortalized cADSCs on CD4^+^ Th cells from mice with DSS-induced colitis. (**A**) Gating strategy for cell proliferation analysis by flow cytometry. Red boxes indicate gating regions. In the left panel: gating of lymphocyte fractions, removal of doublets and dead cells, gating of CD4-positive cells, and analysis of dividing cells on cell trace violet histograms; (**B**) dot plots and histograms showing the division of CD4^+^ cells in each group. CD4^+^ Th cell proliferation was quantified as the percentage of cell trace violet^low^ cells; (**C**) comparison of proliferative CD4^+^ Th cells stimulated with anti-mouse CD3/CD28 antibodies when co-cultured with primary cADSCs, cADSC-K4DT, and cADSC-K4D. The proliferation of mouse CD4^+^ Th cells was significantly inhibited by co-culture with primary and immortalized cADSCs compared with CD4^+^ Th cells alone stimulated with anti-mouse CD3/CD28 antibodies. The inhibitory effect on mouse CD4^+^ Th cell proliferation was stronger for cADSC-K4DT and cADSC-K4D than primary cADSCs. Data are expressed as the mean ± standard deviation from three independent experiments; *n* = 5; ** *p* < 0.01.

To investigate whether immortalized cADSCs also inhibited Th cell proliferation, immortalized cADSCs at a population doubling level (PDL) of 20 were co-cultured with CD4^+^ Th cells isolated from the spleens of colitic mice stimulated with anti-mouse CD3/CD28 antibodies. Th cell proliferation was then measured by dye dilution using flow cytometry. The proliferation of mouse CD4^+^ Th cells was significantly inhibited by both primary cADSCs (41.7% ± 2.1%) and immortalized cADSCs (cADSC-K4DT: 22.6% ± 3.5%; cADSC-K4D: 19.3% ± 1.2%) compared with mono-culture (Th cells alone; 78.5% ± 5.1%). Furthermore, the inhibitory effect was significantly stronger for immortalized cADSCs than primary cADSCs (Figure 5).

### 2.5. Inhibitory Effect of Immortalized cADSCs on Canine T Cell Proliferation

**Figure 6:**
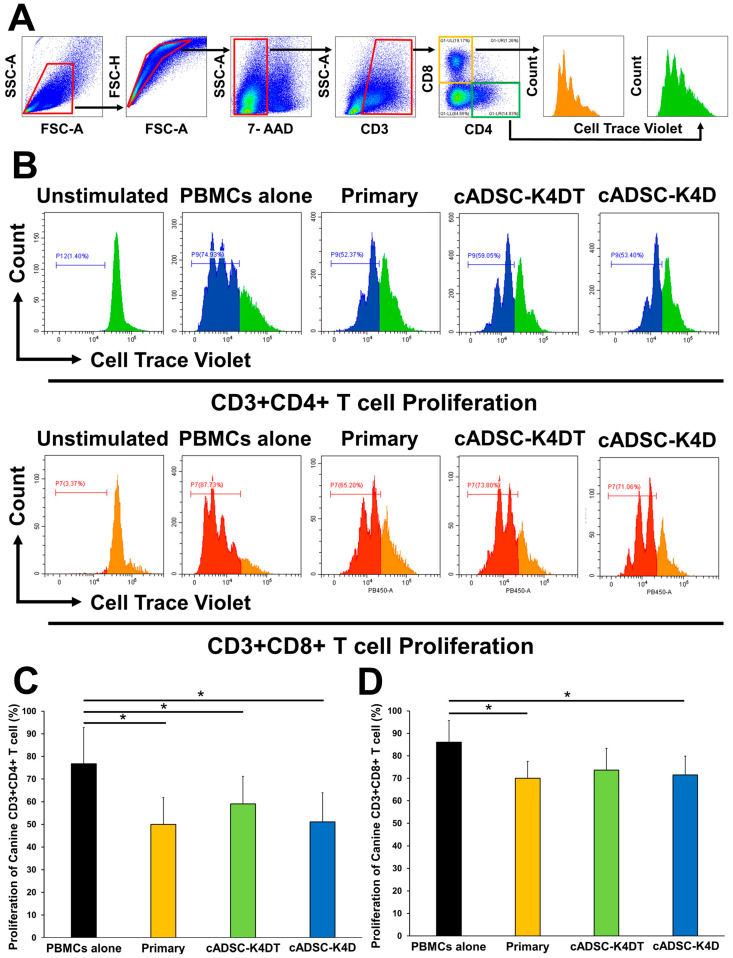
Proliferation inhibitory effect of immortalized cADSCs on canine T cells. (**A**) Gating strategy consisted of gating lymphocyte fractions, doublet and dead cell removal (red boxes), gating CD3^+^, CD4^+^ (green box), or CD8^+^ cells (yellow box), and analysis of CD3^+^CD4^+^ and CD3^+^CD8^+^ dividing cells on a cell trace violet histogram; (**B**) histograms showing the division of CD3^+^CD4^+^ Th cells (upper) and CD3^+^CD8^+^ cytotoxic T lymphocytes (CTLs; lower) in each group. T cell proliferation was quantified as the percentage of cell trace violet^low^ cells; (**C**) comparison of proliferative CD3^+^CD4^+^ Th cells stimulated with ConA when co-cultured with primary cADSCs, cADSC-K4DT, and cADSC-K4D. Co-culture with primary or immortalized cADSCs significantly inhibited canine CD3^+^CD4^+^ Th cell proliferation in all groups; (**D**) comparison of proliferative CD3^+^CD8^+^ CTLs stimulated with ConA when co-cultured with primary or immortalized cADSCs. Primary cADSCs and cADSC-K4D significantly inhibited CD3^+^CD8^+^ CTL proliferation. Data are expressed as the mean ± standard deviation from three independent experiments; *n* = 5; * *p* < 0.05.

To determine whether immortalized cADSCs also exerted allogeneic immunosuppressive effects on canine T cells, they were co-cultured with concanavalin A (ConA)-stimulated canine peripheral blood mononuclear cells (PBMCs), and then the proliferative capacity of CD3^+^CD4^+^ Th cells and CD3^+^CD8^+^ cytotoxic T lymphocytes (CTLs) among PBMCs was evaluated. The proliferation of canine CD3^+^CD4^+^ Th cells was significantly suppressed when co-cultured with primary cADSCs (49.9% ± 11.8%), cADSC-K4DT (59.1% ± 12.2%), or cADSC-K4D (51.1% ± 12.8%) compared with mono-culture (PBMCs alone; 76.8% ± 16.0%; Figure 6B,C). Similarly, for canine CD3^+^CD8^+^ CTLs, co-culturing with primary cADSCs (70.0% ± 7.5%), cADSC-K4DT (73.7% ± 9.6%), or cADSC-K4D (71.5% ± 8.4%) inhibited proliferation compared with mono-culture (PBMCs alone; 86.0% ± 9.7%), and the inhibition was statistically significant, except for cADSC-K4DT (*p* = 0.06; Figure 6B,D).

## 3. Discussion

Immunomodulatory capacity is one of the most important properties that contributes to the therapeutic efficacy of MSCs. Animal models of various diseases, such as GVHD [37], systemic lupus erythematosus (SLE) [38], asthma [39], and rheumatoid arthritis [40], have been used to assess the immunomodulatory capacity of MSCs in vivo [41]. In particular, the DSS-induced colitis model has been the subject of a great deal of research regarding the immunomodulatory potential and therapeutic effects of MSCs [42]. MSCs are expected to be a novel therapeutic approach not only for refractory Crohn’s disease and ulcerative colitis in humans [43] but also for refractory IBD in dogs and cats [6]. Therefore, this animal model was employed in this study to investigate whether novel immortalized cADSCs maintain their immunomodulatory capacity in vivo and exert therapeutic effects. Although the detailed mechanism has not been elucidated, it is known that DSS is toxic to intestinal epithelial cells by binding to medium-chain-length fatty acids in the colon. Administering DSS via drinking water to mice for several to 10 days causes acute injury with partial crypt depletion on the distal colon, together with multiple mucosal erosive lesions with inflammatory cell infiltration [44,45]. Mice that develop acute colitis exhibit gross shortening of the colon and symptoms of weight loss, diarrhea, and rectal bleeding. In DSS-induced colitis, MSCs ameliorate symptoms and histopathological injury by immunomodulation [46,47] and tissue repair [48,49] through various mechanisms of action. In the present study, our immortalized cADSCs significantly suppressed the body weight loss and elevation of the DAI in DSS-induced colitic mice equivalent to primary cADSCs, which confirmed their therapeutic efficacy in vivo. This suggests that the immortalized cADSCs maintained the functional features of primary cADSCs, which contributed to their therapeutic efficacy against DSS-induced colitis. However, we observed no significant improvement in colon length or histology. Compared with previous studies in which cADSCs were administered to mice with DSS-induced colitis using the same experimental design in this study [50], the body weight loss, DAI, colon length, and histological scores in mice with DSS-induced colitis in the PBS group without cADSC treatment in this study were all worse, indicating that the severity of colitis in mice was higher and may, therefore, have masked the effect of cADSCs on the colon length and histology. Differences in the rearing environment and gut microbiota may have contributed to this difference in severity. Moreover, reducing the concentration and duration of DSS administration may have reduced the severity of colitis and accentuated the therapeutic effect of cADSCs but could have led to variations in severity among mice. Because this study was designed to evaluate the equivalence and not the superiority of the therapeutic effects of the immortalized and primary cADSCs, it was reasonable to use mice with reproducible colitis that exhibited consistent severity.

Macrophages are important targets for the immunomodulatory actions of MSCs. In the context of inflammatory diseases, MSCs exert their anti-inflammatory effects by shifting proinflammatory classically activated M1 macrophages to anti-inflammatory alternatively activated M2 macrophages through the paracrine effects of secreted factors, such as prostaglandin E2 (PGE2) [51,52], transforming growth factor (TGF)-β [53,54], and IL-10 [55], and direct cell contact via intercellular adhesion molecule-1 and lymphocyte function-associated antigen-1 [56]. This suppresses T cell activation and induces Treg cells. In the gastrointestinal tract, which is constantly exposed to foreign antigens, such as bacteria, viruses, and food, macrophages play an important role at the forefront of biological defense and regulate the barrier function of the epithelial layer [57]. In the acute phase of DSS-induced colitis when the colonic epithelial barrier is disrupted by DSS, macrophages are stimulated to polarize the M1 phenotype and eliminate pathogens by producing nitric oxide and secreting large amounts of proinflammatory cytokines, such as tumor necrosis factor (TNF)-α, IL-1β, and IL-6, but cause direct colonic mucosal injury and exacerbate colitis [58,59]. MSCs improve gastrointestinal inflammation and the severity of colitis by suppressing the polarization of M1 macrophages in the colon, mesenteric lymph nodes, spleen, and peritoneum, and shifting them to the M2 phenotype through the cyclooxygenase (COX)-2/PGE2 pathway [60,61], TNF-α induced gene/protein 6 (TSG-6) production [62], and microRNA transmission [63]. Similarly, cADSCs and their extracellular vesicles stimulated by inflammatory cytokines shift the colon, spleen, and peritoneal macrophages of DSS-induced colitic mice from the M1 to M2 phenotype via the COX-2/PGE2 pathway [64,65]. In this study, immortalized cADSCs significantly reduced the increase in peritoneal M1 macrophages, while significantly increasing M2 macrophages, and this effect was comparable with primary cADSCs. These results suggest that immortalized cADSCs maintain the abovementioned regulatory mechanisms of macrophages, although further expression and functional analyses of PGE2 and TSG-6 in immortalized cADSCs and their extracellular vesicles are needed.

T cells play a central role in adaptive immunity, and activated effector T cells eliminate pathogens, such as viruses and bacteria. CD4^+^ Th cells, an effector T cell subset, are further classified into many subsets by their cytokine profiles, which are critical for proper immune cell homeostasis and host defense but are also strongly involved in the pathogenesis of autoimmune and inflammatory diseases [66]. Th1 cells, defined by the production of IFN-γ and the expression of the transcription factor T-bet, are involved in cell-mediated immunity and are important for immunity against intracellular pathogens while being associated with the pathogenesis of autoimmune diseases such as SLE, multiple sclerosis, and rheumatoid arthritis [67]. Th2 cells, defined by the production of IL-4 and the expression of the transcription factor GATA-3, are involved in humoral immunity and eliminate extracellular pathogens but are associated with the development of allergic diseases, such as allergic rhinitis [68] and asthma [69]. Additionally, recent studies have identified Th17 cells that produce large amounts of IL-17 and express RORγt, which have been implicated in the pathogenesis of multiple autoimmune and inflammatory diseases [70,71,72]. Many reports have shown that MSCs ameliorate disease by regulating the balance of Th cells in response to pathological conditions [3,41]. For example, MSCs suppress Th1 cell responses in autoimmune diseases, such as acute GVHD [73] and SLE [74], while causing a shift from the Th2 to Th1 cell response in airway allergic inflammatory diseases, such as allergic rhinitis [75] and asthma [76]. Although differences exist in immune responses among species and strains [35,77], in the context of the acute phase of DSS-induced colitis in C57BL/6J mice, Th1/Th17 cell responses characterized by cytokine profiles, such as IFN-γ, TNF-α, IL-6, and IL-17, have been reported to be involved in the disease pathogenesis [35,78]. MSCs ameliorate DSS-induced colitis by suppressing Th1/Th17 cell responses and inducing Treg cells either by direct or indirect actions through the induction of M2 macrophages, which is mediated by PGE2 [79], TSG-6 [80,81], and the Fas ligand [47]. The immortalized cADSCs in this study also decreased the frequency of Th1 and Th17 cells and significantly increased Treg cells together with an increase in M2 macrophages, indicating that they exerted their expected immunomodulatory effects on DSS-induced colitis. Interestingly, this capacity to regulate the Th cell balance was more evident for immortalized cADSCs than primary cADSCs. The reasons for this are unknown, but designing studies to investigate the mechanisms by which immortalized cADSCs modify the CD4^+^ Th cell phenotype is warranted, in addition to elucidating their mechanism of action in macrophages.

Inhibition of T cell proliferation is the most fundamental and important action of MSCs in terms of their immunomodulatory capacity and has been the subject of numerous studies [3,82]. MSCs inhibit T cell proliferation through direct cell contact and secretion of soluble factors, but the mechanism varies by tissue source and animal species [83]. For example, human MSCs inhibit lymphocyte proliferation through the production of indoleamine 2,3-dioxygenase, whereas nitric oxide plays a role in mouse MSCs [84]. In this regard, TGF-β [85,86], adenosine receptor [85], and indoleamine 2,3-dioxygenase [86] appear to be important mediators of the inhibitory effect of cADSCs on lymphocyte proliferation. Additionally, canine bone marrow-derived MSCs (BMSCs) [87], human embryonic stem cell-derived MSCs [88], and equine umbilical cord-derived MSCs [89] inhibit the proliferation of both canine CD4^+^ Th cells and CD8^+^ CTLs, but it is unknown whether cADSCs inhibit CTL proliferation. Therefore, in this study, we investigated whether primary and immortalized cADSCs modulated the Th cell balance in vivo and inhibited the proliferation of Th cells from mice with DSS-induced colitis in vitro and whether they inhibited canine CD4^+^ Th cell and CD8^+^ CTL proliferation in vitro. The results showed that both primary and immortalized cADSCs inhibited the proliferation of Th cells from mice with colitis and canine CD4^+^ Th cell and CD8^+^ CTL proliferation. The inhibitory effect was somewhat weaker on CD8^+^ CTLs than other cell types, probably because of the stronger stimulatory effect of ConA on CD8^+^ CTLs. Taken together with their ability to shift macrophage polarity and Th cells to an anti-inflammatory phenotype and their capacity to inhibit T cell proliferation, we conclude that our immortalized cADSCs maintained the immunomodulatory capacity of primary cADSCs, especially cADSC-K4D, which may be more promising than cADSC-K4DT. In our previous study, cADSC-K4D also had a higher proliferative capacity than cADSC-K4DT without the loss of CD90 with passaging and a higher capacity to inhibit canine PBMC proliferation [14]. In the future, we will further analyze the function of cADSC-K4D at the molecular level and its subpopulations.

There are several limitations to this study that should be noted. One of them was the use of only one experimental model to assess the immunomodulatory capacity of immortalized cADSCs. The DSS-induced colitis model used in this study has been the most used preclinical study of MSC-based therapy for IBD [42]. However, this model may be insufficient to assess the immunomodulatory capacity of MSCs for adaptive immune responses driven by T cells, as severe injury of the intestinal epithelium by DSS is followed by secondary inflammation mediated by innate immune cells [58,59]. Therefore, it is important to simultaneously examine colitis models induced by trinitrobenzene sulfonic acid, which injures the mucosal barrier and acts as a hapten for the gut microbiota, causing Th1-dependent immune activation and infiltration of CD4^+^ T cells into the intestinal mucosa, or oxazolone, which causes Th2 cell responses, in order to provide more insight into the immunomodulatory potential and efficacy of immortalized cADSCs [90]. The cADSC dosing scheme may also have affected the results. In this study, cADSCs were administered by a single intraperitoneal injection of 2 × 10^6^ cells, a common method of MSC administration for DSS-induced colitis. In this regard, a study in which 1 × 10^6^ cells of human tonsil-derived MSCs were injected intraperitoneally twice or four times showed that four repeated doses had a higher therapeutic effect [91]. The route of administration can also alter the biodistribution of MSCs and affect therapeutic efficacy. Wang et al. reported that intraperitoneal injection of mouse BMSCs was more effective and immunomodulatory than intravenous administration [92], while other studies have shown that intravenous administration of mouse ADSCs was more anti-inflammatory than intraperitoneal injection [93]. Thus, different dosing schemes may have better defined the therapeutic effect of our cell line on DSS-induced colitis. Finally, quantification of colonic cytokines and investigation of immune cells in colon tissue by immunohistochemistry are also needed to determine the local immune status of the colon rather than systemic immune status. Such studies should further clarify the efficacy and immunomodulatory properties of immortalized cADSCs.

## 4. Materials and Methods

### 4.1. Preparation of Primary and Immortalized cADSCs

Primary cADSCs were isolated from three healthy beagles (males; mean age: 1.5 years; mean weight: 10.3 kg), and immortalized cADSCs were generated by transduction of canine *TERT*, canine *CCND1*, and human *CDK4R24C* via lentiviral vectors as described previously [14]. Two types of immortalized cADSCs were generated: cells transduced with human *CDK4R24C*, canine *CCND1*, and canine *TERT* (cADSC-K4DT), and cells transduced with human *CDK4R24C* and canine *CCND1* (cADSC-K4D). In this study, primary cADSCs at passage 3 and immortalized cADSCs at PDL 20 were used for all experiments. The animal study protocol was approved by the Bioethics Committee of Nippon Veterinary and Life Science University (approval number 2022S-47, 17 March 2023). The dogs and mice were handled in accordance with the animal care guidelines of the Institute of Laboratory Animal Resources, Nippon Veterinary and Life Science University, Japan.

### 4.2. Characterization of Primary and Immortalized cADSCs

Primary cADSCs and cADSC-K4DT and cADSC-K4D were characterized by the expression of surface markers, such as CD29, CD34, CD44, CD45, CD90, and HLA-DR, by flow cytometry before experiments (Supplementary Figure S1 and Table S1). Additionally, the differentiation potential of these cells was confirmed with respect to adipogenesis, osteogenesis, and chondrogenesis (Supplementary Figure S2).

### 4.3. Animal Experiments

The in vivo immunomodulatory effects of immortalized cADSCs were investigated by experiments in mice with DSS-induced colitis. Thirty 6-week-old male C57BL/6J mice were purchased from Jackson Laboratory Japan (Kanagawa, Japan) and housed in a temperature- and light-controlled (12 h light/dark cycle) room with free access to sterile water and standard laboratory chow. The mice were treated with 3% DSS (36–50 kDa; MP Biomedical, Solon, OH, USA) in the drinking water from day 1 to 7, followed by sterile water administration from day 8 to 10 to induce colitis, whereas the healthy control group received sterile water from day 1 to 10. The mice were randomly divided into four groups of five mice per group and intraperitoneally administered PBS, primary cADSCs, or cADSC-K4DT or cADSC-K4D on day 2 after DSS treatment. cADSCs suspended in 200 μL PBS were injected at a dose of 2 × 10^6^ cells into mice with colitis, whereas the control and PBS groups received the same volume of PBS. On day 10, the mice were sacrificed, and cells and tissues were collected.

### 4.4. Evaluation of DSS-induced Colitis Severity

To evaluate the colitis severity, body weight and the stool condition were monitored daily for 9 days. The DAI was calculated as the sum of three scores: body weight loss relative to initial weight (grades 0–4: 0, none; 1, 1–5% loss; 2, 6–10% loss, 3, 11–20% loss; 4, >20% loss), stool consistency (grades 0–3: 0, normal; 1, soft; 2, loose; 3, watery), and rectal bleeding (grades 0–3: 0, none; 1, occult blood; 2, visible bleeding; 3, gross bleeding). Additionally, colon tissue was collected on day 10 and measured for length.

### 4.5. Histological Analysis

For histological assessment, collected colon tissue was fixed in a 10% formalin solution, embedded in paraffin, sectioned at 3 μm thicknesses, and then stained with hematoxylin and eosin (H&E). Histological examination was performed blindly by two pathologists. The histological score was calculated as the sum of two scores as reported previously [65]: inflammatory cell infiltration (score 0–4: 0, no filtration; 1, infiltrate around crypt basis; 2, infiltrate reaching the lamina muscularis mucosa with abundant edema; 3, extensive infiltration reaching the muscularis mucosa with abundant edema; 4, infiltration of the submucosa layer) and epithelial damage (score 0–4: 0, normal morphology; 1, loss of goblet cells; 2, loss of goblet cells in large areas; 3, loss of crypts; 4, loss of crypts in large areas). Two slides from each section of the colon were evaluated per mouse, and three areas in each slide were examined.

### 4.6. Isolation and Phenotypic Analysis of Mouse Peritoneal Macrophages

To investigate the effect of immortalized cADSCs on macrophage polarization, mouse peritoneal macrophages were isolated. After injecting 4 mL cold PBS into the abdominal cavity of anesthetized mice and gently massaging the abdomen with a finger for 1 min, as much peritoneal lavage fluid as possible was collected with a syringe and centrifuged at 400× *g* for 5 min at 4 °C. To remove erythrocytes, the pellet was suspended in 1 mL RBC lysis buffer (BioLegend, San Diego, CA, USA) and incubated on ice for 5 min, followed by the addition of 5 mL cold PBS to stop the reaction. The sample was centrifuged at 400× *g* for 5 min at 4 °C, and the pellet was washed in 10 mL cold PBS. The pellet was resuspended in 1 mL RPMI 1640 medium supplemented with 10% fetal bovine serum (FBS; Capricorn, Hessen, Germany), 1% antibiotic–antimycotic solution (Thermo Fisher Scientific, Waltham, MA, USA), 1% nonessential amino acids, and 50 μM 2-mercaptoethanol, seeded at 1 mL/well in 6-well culture plates, and incubated at 37 °C overnight. The cells were then washed three times with 1 mL PBS to remove floating cells, and the adherent cells were detached using Accumax (Stemcell Technologies, Vancouver, BC, Canada) to obtain peritoneal macrophages. Subsequently, the cells were washed twice with FACS buffer (PBS with 2% FBS) and incubated with a mouse Fc receptor binding inhibitor (BioLegend, San Diego, CA, USA) on ice for 10 min. After Fc blocking, the macrophages were stained with anti-F4/80-FITC (clone: BM8; BioLegend, San Diego, CA, USA), anti-CD80-APC (clone: 16-10A1; BioLegend, San Diego, CA, USA), and anti-CD206-PE (clone: C068C2; BioLegend, San Diego, CA, USA) antibodies or their respective isotype control, followed by flow cytometry using a CytoFLEX instrument (Beckman Coulter, Brea, CA, USA) and data analysis with CytExpert ver 2.0 analysis software.

### 4.7. Isolation and Phenotypic Analysis of Mouse Splenic CD4^+^ Th Cells

For immunophenotypic analysis of splenic CD4^+^ Th cells from mice after treatment and to examine the ability of immortalized cADSCs to inhibit CD4^+^ Th cell proliferation, spleens were collected from mice, minced into small pieces on ice in RPMI 1640 medium, and mashed with the plunger end of a syringe on a 70 μm cell strainer. A cell suspension was then obtained by washing the cell strainer with 5 mL RPMI 1640 medium. After centrifuging the cell suspension at 400× *g* for 5 min at 4 °C, the pellet was suspended in 5 mL RBC lysis buffer and incubated on ice for 5 min to lyse erythrocytes. The reaction was stopped by adding 25 mL cold PBS, and then the sample was centrifuged at 400× *g* for 5 min at 4 °C. The pellet was washed twice with 10 mL cold PBS and stained with trypan blue to count the cells. From the single-cell suspension of splenic mononuclear cells, CD4^+^ Th cells were purified by negative magnetic selection using a mouse CD4^+^ T cell isolation kit (Miltenyi Biotec, Bergisch Gladbach, Germany) in accordance with the manufacturer’s instructions. The purity of CD4^+^ Th cells after purification was >90%.

To analyze Treg cells, isolated CD4^+^ Th cells were immediately washed with FACS buffer, incubated with the mouse Fc receptor binding inhibitor on ice for 10 minutes, and then stained with anti-CD4-APC (Clone: RM4-5; BioLegend, San Diego, CA, USA) and anti-CD25-PE (clone: PC61; BioLegend, San Diego, CA, USA) antibodies or their respective isotype controls. Subsequently, the cells were fixed and permeabilized using True-Nuclear Transcription Factor Buffer Set (BioLegend, San Diego, CA, USA) and stained with an anti-Foxp3-Alexa Fluor488 antibody (clone: MF-14; BioLegend, San Diego, CA, USA) or isotype control. To analyze Th cell polarization, isolated CD4^+^ Th cells were stimulated with phorbol 12-myristate 13-acetate (50 ng/mL, Sigma-Aldrich, St. Louis, MO, USA) and ionomycin (1 μg/mL, Sigma-Aldrich, St. Louis, MO, USA) for 6 h and brefeldin A (10 μg/mL, Sigma-Aldrich, St. Louis, MO, USA) for 4 h. After stimulation, the cells were collected, washed with FACS buffer, incubated with the mouse Fc receptor binding inhibitor on ice for 10 minutes, and then stained with an anti-CD4-APC antibody (Clone: RM4-5; BioLegend, San Diego, CA, USA) or isotype control, fixed, and permeabilized using Cyto-Fast Fix/Perm Buffer Set (BioLegend). The cells were stained with anti-IFN-γ-PE (clone: XMG1.2; BioLegend, San Diego, CA, USA), anti-IL-4-PE (clone: 11B11; BioLegend, San Diego, CA, USA), or anti-IL-17A-PE (clone: TC11-18H10.1; BioLegend, San Diego, CA, USA) antibodies, or their respective isotype controls. Analysis by flow cytometry was performed by measuring the frequency of cells expressing IFN-γ, IL-4, or IL-17A among gated CD4^+^ cells and the frequency of cells expressing Foxp3 among gated CD4^+^CD25^+^ cells.

### 4.8. Isolation of Canine PBMCs

Blood was collected from the jugular vein of five healthy adult beagles into heparinized tubes, followed by immediate separation of PBMCs by density gradient centrifugation using Histopaque-1077 (Sigma-Aldrich, St. Louis, MO, USA) and SepMate-15 (VERITAS, Tokyo, Japan). Isolated PBMCs were resuspended in RPMI 1640 medium supplemented with 10% FBS, 1% antibiotic-antifungal solution, 1% nonessential amino acids, and 50 μM 2-mercaptoethanol for further experiments.

### 4.9. Lymphocyte Proliferation Assay

To evaluate the immunosuppressive capacity of immortalized cADSCs in vitro, mouse splenic CD4^+^ Th cells or canine PBMCs were co-cultured with immortalized cADSCs. Isolated mouse CD4^+^ Th cells or canine PBMCs were pre-labeled with a 5 μM CellTrace Violet solution using a CellTrace Violet Cell Proliferation Kit (Thermo Fisher Scientific, Waltham, MA, USA) in accordance with the manufacturer’s instructions before seeding and added at 1 × 10^6^ cells to wells with or without 2 × 10^5^ primary cADSCs, cADSC-K4DT, or cADSC-K4D (*n* = 5 per group). Mouse CD4^+^ Th cells were then stimulated with anti-mouse CD3/CD28 antibody-loaded Anti-Biotin MACSiBead particles using a mouse T Cell Activation/Expansion Kit (Miltenyi Biotec, Bergisch Gladbach, Germany) in accordance with the manufacturer’s instructions, and canine PBMCs were activated with 5 μg/mL ConA and maintained at 37 °C with 5% CO_2_ for 72 h. After stimulation, the cells were collected and washed with FACS buffer. Then, mouse CD4^+^ Th cells were incubated with the mouse Fc receptor binding inhibitor, whereas canine PBMCs were incubated with a canine Fc receptor binding inhibitor (Thermo Fisher Scientific, Waltham, MA, USA) on ice for 20 minutes. Subsequently, the mouse CD4^+^ Th cells were stained with an anti-CD4-APC antibody or the isotype control and the canine PBMCs were stained with anti-CD3-FITC (Clone: CA17.2A12; Bio-Rad, Tokyo, Japan), anti-CD4-RPE (Clone: YKIX302.9; Bio-Rad, Tokyo, Japan), and anti-CD8-Alexa Fluor 647 (Clone: YCATE55.9; Bio-Rad, Tokyo, Japan) antibodies or their respective isotype controls. To remove dead cells from the analysis, nuclei were stained with 7-amino-actinomycin D (BioLegend, San Diego, CA, USA). The proliferation of mouse CD4^+^ Th cells, CD3^+^CD4^+^, and CD3^+^CD8^+^ T cells among canine PBMCs was measured by flow cytometry.

### 4.10. Statistical Analysis

Data are presented as the mean ± standard deviation. Differences among multiple groups were assessed by one-way analysis of variance and compared using the Tukey–Kramer post hoc test. *p* < 0.05 was considered statistically significant. Statistical analyses were performed using R commander 4.1.2.

## 5. Conclusions

Our established immortalized cells, cADSC-K4DT and cADSC-K4D, had therapeutic potential in mice with DSS-induced colitis as a model of inflammatory disease and exerted immunomodulatory effects both in vivo and in vitro. Further investigation of the therapeutic efficacy and safety in other disease models and efforts to elucidate the mechanism of action of immortalized cADSCs may increase the feasibility of stem cell therapy using immortalized cADSCs as a stable cell source.

## Data Availability

The data supporting the findings of this study are available from the corresponding author upon reasonable request.

## Supplementary Materials

The supporting information can be downloaded at https://www.mdpi.com/article/10.3390/ijms242417484/s1.
