# Supplementary material for: Immortalized Canine Adipose-Derived Mesenchymal Stem Cells Maintain the Immunomodulatory Capacity of the Original Primary Cells
Source: Int J Mol Sci. 2023 Dec 14;24(24):17484. doi: 10.3390/ijms242417484 (PMC10743981; doi:10.3390/ijms242417484)
Supplement: Supplementary file 1 [file ijms-24-17484-s001.zip › ijms-2745247-supplementary.pdf]

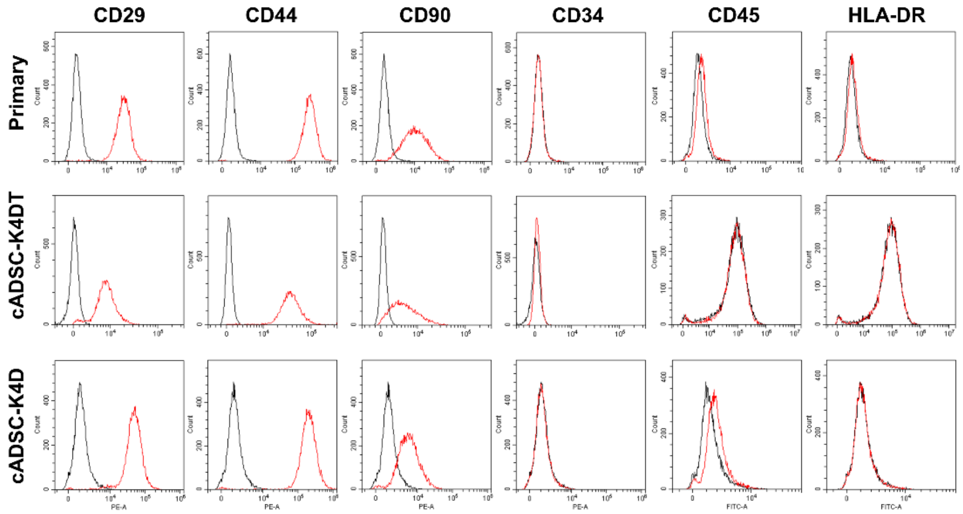

Figure S1. Expression of cell surface markers analyzed by flow cytometry. Primary cADSCs at passage 3; cADSC-K4DT and cADSC-K4D cells at PDL20 are shown. Black lines indicate isotype controls and red lines indicate each cell line.

Table S1. Flow cytometric analysis of cell surface markers.

	CD29	CD44	CD90	CD34	CD45	HLA-DR
Primary	98.5 ± 1.7	97.8 ± 1.5	90.7 ± 1.5	0.5 ± 0.1	0.4 ± 0.2	0.4 ± 0.3
cADSC-K4DT	97.8 ± 1.4	98.2 ± 0.9	79.2 ± 3.2	0.4 ± 0.1	0.4 ± 0.2	0.4 ± 0.2
cADSC-K4D	98.6 ± 1.1	98.5 ± 1.2	85.6 ± 2.1	0.4 ± 0.2	0.3 ± 0.2	0.4 ± 0.2

Data are expressed as the percentage of positive cells (mean ± standard error); n = 3 for three independent experiments.

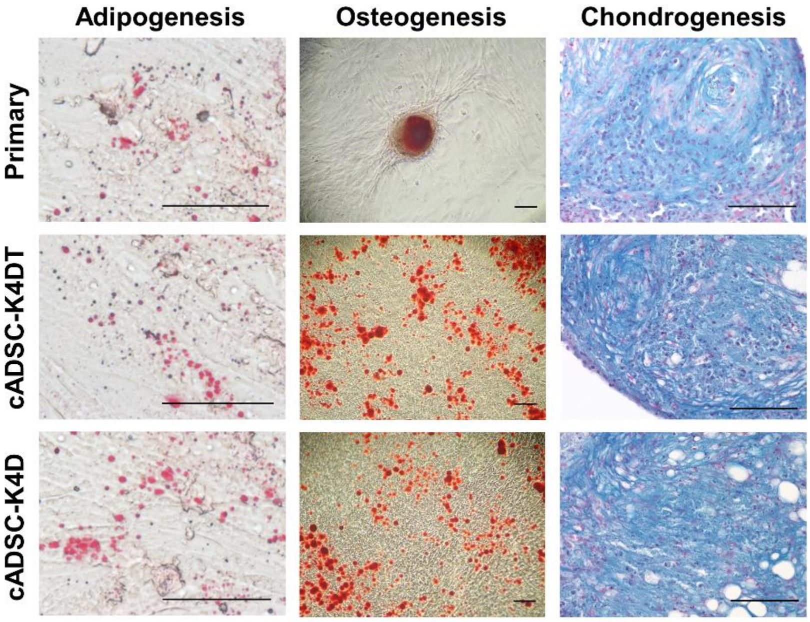

Figure S2. Trilineage differentiation potential of primary cADSCs at passage 3; cADSC-K4DT and cADSC-K4D cells at PDL 20. Adipocytes, osteocytes, and chondrocytes were stained with oil red O, alizarin red, and alcian blue, respectively. Bar = 100 μm.
